# Editorial: Insights in aging and the immune system: 2021

**DOI:** 10.3389/fragi.2022.999299

**Published:** 2022-08-25

**Authors:** Laura Haynes, Leena P. Bharath

**Affiliations:** ^1^ UConn Center on Aging, University of Connecticut School of Medicine, Farmington, CT, United States; ^2^ Department of Nutrition and Public Health, Merrimack College, North Andover, MA, United States

**Keywords:** aging, inflammation, autoimmune disease, senescence, T cells

This Research Topic explores some recent advances in the field of Aging and the Immune System. We asked our Editorial Board members for forward-looking contributions that describe the state of the art, outlining recent developments and accomplishments. Our goal was to shed light on some of the progress made in the past decade in the field of Aging and the Immune System and how this relates to age-related changes in the health of older adults. Collectively, these articles emphasize the point that aging of the immune system is associated with increased inflammation as well as dysregulation of both innate and adaptive immune responses, which can then contribute to the development of many age-associated diseases.

Two original research articles that were contributed examine how aging impacts autoimmunity and anti-tumor responses. In the article *Obesity Accelerates Age-Associated Defects in Human B Cells Through a Metabolic Reprogramming Induced by the Fatty Acid Palmitate*, Frasca et al. examined the concept that obesity and aging may exert similar impacts on the immune system via induction of chronic inflammation. They focused on production of autoantibodies, which are increased with both obesity and aging. They found that fatty acids can reprogram the metabolism of B cells from young lean subjects to function like those from young obese or elderly lean subjects, thus linking the impact of fatty acid-induced inflammation to dysregulation of B cell responses. Duong et al., *Aging Leads to Increased Monocytes and Macrophages with Altered CSF-1 Receptor Expression and Earlier Tumor-associated Macrophage Expansion in Murine Mesothelioma*, examined the response to mesothelioma in young and aged mice in order to understand how aging impacts the response to tumors. They found that Ly6C^high^ macrophages, which have increased proinflammatory responses, are increased in older mice with tumors and prior work has shown that these cells may contribute to diminished anti-tumor activity that occurs with aging.

The mini-review *Growth Differentiation Factor-15 in Immunity and Aging* by Pence examines the immunoregulatory and anti-inflammatory roles of growth differentiation factor-15 (GDF-15). GDF-15 can be a component of the senescence associated secretory phenotype (SASP) and its production is induced in response to proinflammatory conditions in order to dampen inflammation. Importantly, the plasma concentration of GDF-15 increases with aging, likely as a result of chronic age-associated inflammation. This age-related elevation of GDF-15 could also function to dampen immune responses, including those to infectious agents, which could explain why it is one of the proteins most strongly correlated with multimorbidity in older adults. The review article *Inflammation, Immune Senescence, and Dysregulated Immune Regulation in the Elderly*, by Shive and Pandiyan extends the analysis of inflammation and dysregulated responses in this Research Topic by providing an overview of age-related inflammation and how it impacts the immune response in older adults including an examination of inflammatory and anti-inflammatory cytokines and regulatory T cells. In the review article *T Cell Aging-Associated Phenotypes in Autoimmune Disease*, Zhao et al. and her colleagues focus on the adaptive arm of the immune system and discuss T cell aging associated phenotypes (TASP) and how they contribute to two age-related autoimmune diseases: rheumatoid arthritis (RA) and giant cell arteritis (GCA). RA results from premature aging of T cells, leading to excessive production of inflammatory mediators, while GCA results from age-associated loss of control of T cell induced inflammation, including loss of regulatory T cells. Finally, the review *Proteolysis dysfunction in the process of aging and age-related diseases* from Frankowska et al. presents current insights on how aging impacts proteolytic systems. Failure to appropriately regulate proteolysis can result in protein aggregation and chronic inflammation, which can then lead to age-related diseases.

We hope that this Research Topic provides readers with a glimpse of some of the exciting research that is happening in the field of aging and immunity and that it stimulates new ideas and future progress.

